# Shared Decision-Making With Otolaryngologists and Palliative Care Specialists in Oral Cavity Cancer

**DOI:** 10.1001/jamanetworkopen.2025.48557

**Published:** 2025-12-11

**Authors:** Hsien-Liang Huang, Shao-Yi Cheng, Jaw-Shiun Tsai, Hsin-Yin Su, Yun-Ching Lin, Ya-Chun Kang, Shih-Ying Lee, Yi-Wen Chen, Hsin-Jung Lin

**Affiliations:** 1 Department of Family Medicine, National Taiwan University Hospital, Taipei City, Taiwan; 2 Cancer Administration and Coordination center, National Taiwan University Hospital, Taipei City, Taiwan; 3 Department of Nursing, National Taiwan University Hospital, Taipei City, Taiwan

## Abstract

**Importance:**

Advanced oral cavity cancer greatly affects vital functions, physical symptoms, psychological well-being, and social interactions. Early integration of palliative care is essential to address these burdens, yet access often remains limited, with many patients referred only near the end of life.

**Objective:**

To evaluate whether the shared decision-making with otolaryngologists and palliative care specialists (SOP) model is associated with increased palliative care utilization among patients with advanced oral cavity cancer before death.

**Design, Setting, and Participants:**

This prospective cohort study compared outcomes before and after SOP model implementation at an otolaryngology outpatient clinic at a national referral center. Patients with newly diagnosed stage IV oral cavity cancer undergoing concurrent chemoradiotherapy were included. The SOP model was introduced between 2020 and 2021, while patients treated from 2018 to 2019 received usual cancer care. Follow-up duration was 3 years, through December 2024.

**Exposure:**

The SOP model was structured into 3 sequential steps: choice talk, option talk, and decision talk. During choice talk, otolaryngologists introduced treatment and care options. Option talk and decision talk, facilitated by case managers and palliative care specialists, provided detailed discussion on risks, benefits, and alignment with patient values.

**Main Outcomes and Measures:**

The primary outcome was the composite frequency of multidisciplinary palliative care service utilization delivered alongside anticancer treatment before death. Services included consultations with palliative care specialists, social workers, clinical psychologists or chaplains, and physiatrists. Univariate and multivariate linear regression analyses were performed for continuous outcomes, including the composite frequency of multidisciplinary palliative care consultations and the frequency of medical resource utilization.

**Results:**

Among the 430 eligible patients, 110 were analyzed (mean [SD] age, 57.9 [10.7] years; 102 male patients [93%]); 52 were in the SOP group and 58 were in the non-SOP group. The SOP group had significantly higher frequency of multidisciplinary palliative care consultations compared with the non-SOP group (multivariate linear regression, β = 0.49; 95% CI, 0.11-0.87; *P* = .01).

**Conclusions and Relevance:**

In this cohort study of patients with advanced oral cavity cancer, implementation of the SOP model was associated with greater utilization of multidisciplinary palliative care services. Broader adoption of this model may facilitate timely palliative care engagement and improve the quality of end-of-life care in this population.

## Key Points

**Question:**

Is a model of shared decision-making with otolaryngologists and palliative care specialists (SOP) associated with increased use of palliative care among patients with advanced oral cavity cancer?

**Findings:**

In this cohort study with 430 initially eligible patients and 110 included in the final analysis after 3 years, patients in the SOP group received significantly more multidisciplinary palliative care consultations before death than those in the non-SOP group.

**Meaning:**

These findings suggest that the SOP model may improve timely access to palliative care and enhance end-of-life care quality for patients with advanced oral cavity cancer.

## Teaser

This cohort study examines whether a model of shared decision-making with otolaryngologists and palliative care specialists is associated with increased palliative care utilization among patients with advanced oral cavity cancer.

## Introduction

Oral cavity cancer is a major global health concern, with an estimated 389 485 new diagnoses and 188 230 deaths in 2022.^1^ It continues to represent a persistent burden, with incidence trends varying across regions. Rates remain particularly high in Southeast Asia and the Asia-Pacific region, largely because of cultural habits such as betel quid chewing, whereas in some Western countries, incidence has stabilized or declined in parallel with reductions in tobacco use.^2^ However, in countries such as England, Brazil, Denmark, and the Netherlands, incidence continues to rise. In England, for example, oral cavity cancer cases increased by 63% between 1995 and 2011, with annual growth rates of 2.8% among men and 3.0% among women, and projections indicate a further annual increase of 3% up to 2025.^3^ This persistent and growing burden underscores the importance of focusing not only on prevention but also on improving care pathways and timely integration of palliative care for patients whose cancer is diagnosed at advanced stages.

Advanced oral cavity cancer presents substantial challenges because of its impact on essential functions, physical symptoms, psychological well-being, and social interactions. Functional impairments such as dysphagia, speech difficulties, and breathing problems can lead to malnutrition and reduced quality of life. Patients often experience severe pain, fatigue, xerostomia, and communication barriers, while visible disfigurement and loss of function contribute to social isolation and psychological distress.^4,5^ Socioeconomic disparities further influence access to care, and patients with head and neck cancer face an elevated risk of suicide.^6^ Medical interventions often involve frequent hospital admissions and emergency care for airway complications, infections, and bleeding.^5,7^ Early integration of palliative care is crucial to manage symptoms, provide psychosocial support, enhance communication and decision-making, and coordinate multidisciplinary care.^8^ A patient-centered approach can improve quality of life and ensure that care aligns with patients’ values and preferences.^9,10,11^

Patients with advanced oral cavity cancer often receive inadequate palliative care because of multiple barriers. Access to palliative care remains inconsistent, with many patients referred late.^12^ Limited integration between oncology and palliative care teams further contributes to fragmented care, while misconceptions about palliative care as solely end-of-life treatment may lead to reluctance in seeking support.^5^ Communication challenges, a predominant issue in head and neck cancers, exacerbate difficulties in discussing care preferences.^7^ In addition, health care practitioners may lack specialized knowledge of palliative care for head and neck cancers, underestimating patients’ needs. Many of these patients continue to require acute interventions even in their final weeks, making early and well-coordinated palliative care crucial.^13,14^ In Taiwan, patients with advanced disease usually receive combined care, including targeted therapy, chemotherapy, or palliative radiotherapy, from otolaryngologists, oncologists, and radiation oncologists. Referral to palliative care specialists, however, depends on both physician initiation and patient or family willingness, which often delays integration. As a result, palliative care is typically introduced later than other medical treatments and only at a very advanced stage. Addressing these barriers through better integration, education, and proactive referral strategies is essential to ensuring that patients with advanced oral cavity cancer receive the comprehensive care.

Shared decision-making (SDM) is a key component of patient-centered care that facilitates collaborative discussions ensuring that treatment plans align with patients’ values and goals.^15^ Implementing SDM in patients with advanced cancer has shown multiple benefits, including improved communication, increased documentation of care preferences such as do not resuscitate (DNR) orders, and greater satisfaction with advance care planning.^9,16,17^ Moreover, SDM models have been associated with reduced hospital transfers and increased acceptance of palliative care among patients with advanced cancer.^17,18^ Given the complex care needs of patients with advanced oral cavity cancer, integrating SDM into their care pathway may improve decision-making and facilitate earlier palliative care involvement.

**Figure 1.**
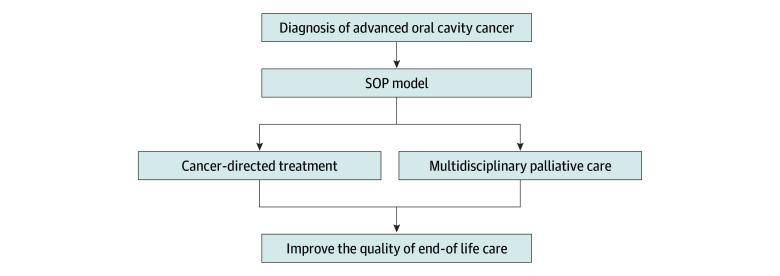
Shared Decision-Making With Otolaryngologist and Palliative Care Specialists (SOP) Model for Patients With Advanced Oral Cavity Cancer

Our team developed the SDM with oncologists and palliative care specialists (SOP) model to help patients with advanced cancer receiving goal concordant care.^9,16,18,19^ This study aimed to implement the SOP model (Figure 1) to promote receipt of palliative care before death among patients with advanced oral cavity cancer.

## Method

### Design and Participants

**Figure 2.**
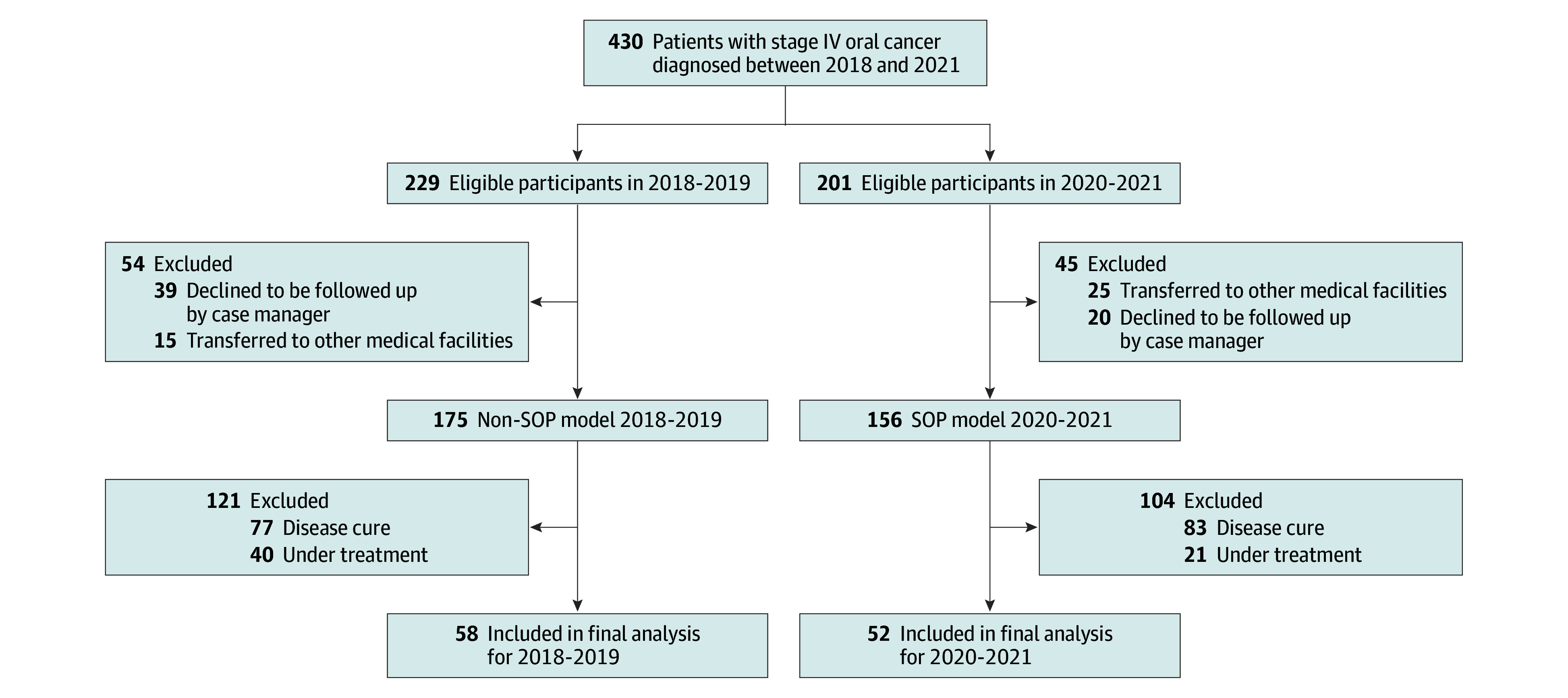
Participant Enrollment Flowchart SOP indicates shared decision-making with otolaryngologist and palliative care specialist.

This cohort study was conducted at an otolaryngology outpatient clinic in a national referral center in Northern Taiwan from January 2018 to December 2024. Participants were eligible to participate if they had a new diagnosis of stage IV oral cavity cancer under concurrent chemoradiotherapy (CCRT), were aged 20 years or older, and were able to read and respond to the decision aid provided in the SOP model. Because the SOP model was fully implemented for patients with advanced oral cavity cancer beginning in 2020, we designated patients treated in 2020 to 2021 as the case (SOP) cohort. Patients treated in 2018 to 2019, before implementation, received usual care with case managers’ follow-up according to the cancer treatment schedule only and served as the control cohort, because their numbers were comparable to those in the SOP group. We did not include patients from 2022 onward because the COVID-19 pandemic markedly altered health care delivery, which could have introduced bias. Each patient in both cohorts was followed up for 3 years and was included in the final analysis if death occurred within this follow-up period (Figure 2). This study was conducted in accordance with the Strengthening the Reporting of Observational Studies in Epidemiology (STROBE) reporting guidelines for cohort studies. Ethical approval was obtained in accordance with the Declaration of Helsinki^20^ from the hospital research ethics committee. The requirement for informed consent from study participants was waived by the research ethics committee.

### SOP Model Development and Intervention

The SOP model used in this study was developed in 2016 by integrating the traditional 3-talk model of SDM.^15,21^ In brief, this model serves as a structured framework to facilitate communication among patients, families, and health care practitioners, supporting informed decision-making regarding treatment and care with a combination of system-related and patient-specific factors. A detailed description of the framework is available elsewhere.^9,16,18,19^ The SOP model was structured into 3 sequential steps: choice talk, option talk, and decision talk. During choice talk, otolaryngologists introduced available treatments and care options for patients with advanced oral cavity cancer and their families. After the otolaryngologist clinic, an oral cavity cancer case manager introduced the SOP model and provided patients with a decision-support pamphlet. This pamphlet, which was designed according to the Grading of Recommendations, Assessment, Development, and Evaluation (GRADE) framework,^22,23^ incorporated quality-of-care principles, and evidence-based medicine. Case managers also facilitated explorations on patient preferences regarding anticancer treatments and palliative care treatments as the option talk phase and prepare patient for the subsequent decision talk in the palliative care specialist clinic. The choice talk phase, provided through otolaryngologists and oral cavity cancer case managers, illustrates system-level improvements in SDM.

The option talk and decision talk phases aimed to clarify the benefits and risks of each treatment and alternative and to highlight improvements at the patient-specific level by ensuring that decisions are aligned with individual values and preferences. The communication process prioritized 3 key objectives: first, establishing rapport; second, addressing symptoms and adverse effects of ongoing anticancer treatments; and third, exploring patient preferences for end-of-life care. Patients were then referred to a multidisciplinary team—including social workers, clinical psychologists, chaplains, physiatrists, and palliative care specialists—on the basis of their decisions.

### Outcome Measures

The primary outcome was the composite frequency of utilization of multidisciplinary palliative care services provided alongside anticancer treatment before death. These services were defined as consultations with 4 categories of medical professionals: palliative care specialists, social workers, clinical psychologists or chaplains, and physiatrists.

Secondary outcomes included a comparison of patient engagement in each of the 4 dimensions of multidisciplinary palliative care consultations, as well as the rate of DNR documentation. Additional outcomes focused on medical resource utilization in the 3 months and 1 month before death, including the frequency of hospital admissions, emergency department visits, intensive care unit admissions, and invasive treatments such as cardiopulmonary resuscitation and tracheostomy with mechanical ventilation.

### Statistical Analysis

Categorical variables including demographic characteristics were presented as proportions and analyzed using the χ^2^ test. Continuous demographic variables were analyzed using the independent *t* test. To evaluate the impact of the SOP model, we performed univariate and multivariate linear regression analyses for continuous outcomes, including the composite frequency of multidisciplinary palliative care consultations and the frequency of medical resource utilization. For categorical outcomes, such as receipt of consultations across each of the 4 dimensions of multidisciplinary palliative care and rates of DNR documentation, we applied univariate and multivariate logistic regression analyses. In all models, the SOP model was treated as the independent variable, whereas outcomes including palliative care consultations, resource utilization, and DNR documentation served as dependent variables. Multivariate analyses were adjusted for sex, age, education level, religion, and marital status to account for potential confounding factors. Two-tailed *P* < .05 was considered statistically significant. All analyses were performed using SPSS statistical software version 21.0 (IBM Corp).

## Results

A total of 430 participants were enrolled between 2018 and 2021. All patients with newly diagnosed stage IV oral cavity cancer preparing for CCRT at our institution were eligible. From 2018 to 2019, 229 patients met the eligibility criteria; after excluding 15 who were transferred to local or regional hospitals for CCRT or subsequent treatment and 39 who were lost to follow-up, 175 patients remained and were designated as the control group. From 2020 to 2021, 201 patients were initially eligible; after excluding 25 who were transferred to local or regional hospitals for CCRT or subsequent treatment and 20 who were lost to follow-up, 156 patients were retained and comprised the SOP group. Following 3 years of observation, and after excluding patients who were cured or still receiving treatment, 58 patients in the control group (2018-2019) had died by December 2022, and 52 patients in the SOP group (2020-2021) had died by December 2024 (Figure 2).

Among the total 110 patients included in the final analysis, the mean (SD) age was 57.9 (10.7) years (58.3 [10.7] years in the SOP group and 57.4 [10.8] years in the non-SOP group). The participants were predominantly male, with 48 (92%) in the SOP group and 54 (93%) in the non-SOP group (total, 102 men [93%]). With regard to education background, most of the participants had a high school education or less (42 participants [81%] in the SOP group and 44 [76%] in the non-SOP group). Most of the participants were married (33 [64%] in the SOP group and 44 [76%] in the non-SOP group). The most common religions in both groups were folklore religion (7 patients [14%] in the SOP group and 11 patients [19%] in the non-SOP group), Buddhism (12 patients [23%] in the SOP group and 16 patients [28%] in the non-SOP group), and Taoism (15 patients [29%] in the SOP group and 15 patients [26%] in the non-SOP group). The χ^2^ analysis showed the homogeneity of the 2 groups of patients (Table 1).

**Table 1.** Demographic Characteristics of Patients

Clinical characteristic	Patients, No. (%)			*P* value^a^
	Overall (N = 110)	SOP (n = 52)	Non-SOP (n = 58)	
Age, mean (SD), y	57.9 (0.7)	58.3 (10.7)	57.4 (10.8)	.66
Sex				
Male	102 (93)	48 (92)	54 (93)	.87
Female	8 (7)	4 (8)	4 (7)	
Education				
Elementary school or below	24 (22)	8 (15)	16 (28)	.34
Junior high school	33 (30)	18 (35)	15 (26)	
Senior high school	29 (26)	16 (31)	13 (22)	
Junior college	14 (13)	6 (12)	8 (14)	
University or above	9 (8)	4 (8)	5 (9)	
Other or unknown	1 (1)	0	1 (2)	
Marital status				
Married	77 (70)	33 (64)	44 (76)	.51
Widowed	5 (5)	3 (6)	2 (3)	
Single	17 (16)	9 (17)	8 (14)	
Divorced	11 (10)	7 (14)	4 (7)	
Other or unknown	0	0	0	
Religion				
Folklore religion	18 (16)	7 (14)	11 (19)	.87
Buddhism	28 (26)	12 (23)	16 (28)	
Taoism	30 (27)	15 (29)	15 (26)	
Christianity	2 (2)	1 (2)	1 (2)	
Not specified	32 (29)	17 (33)	15 (26)	
Other or unknown	0	0	0	

Abbreviation: SOP, shared decision-making with otolaryngologist and palliative care.

^a^ *P* values were calculated using independent *t* test for age and χ^2^ test for the remaining variables.

The primary outcome showed that the SOP group had significantly more frequent utilization of multidisciplinary palliative care services compared with the non-SOP group, as demonstrated in the multivariate linear regression analysis (β, 0.49; 95% CI, 0.11 to 0.87; *P* = .01) (Table 2). In addition, the SOP group had significantly fewer hospital admissions in the 3 months before death (β, −0.53; 95% CI, −1.02 to −0.03; *P* = .04). Regarding emergency department visits 1 month before death, a significant difference was observed in the univariate analysis (β, −0.35; 95% CI, −0.68 to −0.01; *P* = .04), whereas the multivariate model showed a nonsignificant result (β, −0.34; 95% CI, −0.68 to 0.00; *P* = .05).

**Table 2.** Association of the Shared Decision-Making With Otolaryngologist and Palliative Care Specialist Model With Multidisciplinary Palliative Care Consultations and Medical Resource Utilization

Outcome variable	Univariate analysis		Multivariate analysis^a^	
	β (95% CI)	*P* value	β (95% CI)	*P* value
Multidisciplinary palliative care consultations	0.45 (0.07 to 0.84)	.02^b^	0.49 (0.11 to 0.87)	.01^b^
Medical resources utilization 3 mo before death				
Hospital admissions	−0.53 (−1.03 to −0.02)	.04^b^	−0.53 (−1.02 to −0.03)	.04^b^
ED visits	−0.10 (−0.62 to 0.42)	.70	−0.06 (−0.57 to 0.46)	.83
ICU admissions	0.11 (−0.15 to 0.37)	.40	0.12 (−0.14 to 0.38)	.38
Invasive treatments (CPR, tracheostomy with ventilator)	0.10 (−0.17 to 0.36)	.47	0.09 (−0.18 to 0.36)	.51
Medical resources utilization 1 mo before death				
Hospital admissions	−0.16 (−0.38 to 0.06)	.15	−0.16 (−0.38 to 0.07)	.16
ED visits	−0.35 (−0.68 to −0.01)	.04^b^	−0.34 (−0.68 to 0.00)	.05
ICU admissions	−0.03 (−0.20 to 0.14)	.73	−0.03 (−0.20 to 0.14)	.75
Invasive treatments (CPR, tracheostomy with ventilator)	0.05 (−0.18 to 0.28)	.66	0.04 (−0.19 to 0.27)	.74

Abbreviations: CPR, cardiopulmonary resuscitation; ED, emergency department; ICU, intensive care unit.

^a^ Multivariate models were adjusted for sex, age, education level, religion, and marital status.

^b^ Significant difference was defined as *P* < .05.

As shown in Table 3, the logistic regression analysis of the association between the SOP model and the 4 dimensions of multidisciplinary palliative care and DNR documentation rates revealed that only social worker consultations were significantly higher in the SOP group in univariate analysis (odds ratio, 2.22; 95% CI, 1.02 to 4.80; *P* = .04). However, this association was no longer statistically significant in the multivariate model (odds ratio, 2.29; 95% CI, 0.81 to 6.48; *P* = .12). No other dimensions of palliative care or DNR documentation rates showed significant associations with the SOP model in either analysis.

**Table 3.** Association of the Shared Decision-Making With Otolaryngologist and Palliative Care Specialist Model With 4 Dimensions of Multidisciplinary Palliative Care and Do Not Resuscitate Documentation Rates

Outcome variable	Univariate analysis		Multivariate analysis^a^	
	OR (95% CI)	*P* value	OR (95% CI)	*P* value
Palliative care specialist consultation	1.44 (0.54-3.84)	.47	1.95 (0.59-6.50)	.28
Clinical psychologist or chaplain consultation	1.83 (0.79-4.25)	.16	2.13 (0.79-5.73)	.14
Social worker consultation	2.22 (1.02-4.80)	.04^b^	2.29 (0.81-6.48)	.12
Physiatrist consultation	1.43 (0.66-3.10)	.36	1.76 (0.71-4.40)	.23
Do not resuscitate order documentation	0.70 (0.18-2.75)	.61	0.84 (0.17-4.16)	.83

Abbreviation: OR, odds ratio.

^a^ Multivariate models were adjusted for sex, age, education level, religion, and marital status.

^b^ Significant difference was defined as *P* < .05.

## Discussion

Our cohort study found that the SOP model was associated with a higher number of multidisciplinary care consultations for patients with advanced oral cavity cancer compared with the non-SOP group. Because patients were followed up for only 3 years, with a large proportion still receiving treatment, longer follow-up with more outcomes available would provide greater robustness to the findings. Early integration of the SDM approach may help address the prevalent misconception that palliative care is equivalent to giving up or is solely intended for the terminal phase of illness. In contrast, when initiated early in the disease trajectory, palliative care provides comprehensive supportive interventions delivered by a multidisciplinary team to complement ongoing cancer treatment. Common functional impairments in advanced oral cavity cancer, such as dysphagia, speech difficulties, and respiratory compromise, can be more effectively managed through timely palliative care consultations. The SOP model facilitates collaborative engagement between otolaryngologists and palliative care specialists, enhancing patient and family understanding of the purpose and benefits of palliative care, and thereby reducing hesitancy and improving access to these essential services. Beyond clinical practice, our findings suggest that the SOP model could be systematically incorporated into cancer care pathways, with training provided for otolaryngologists, oncologists, and allied professionals to strengthen their skills in SDM. At the policy level, health authorities and stakeholders should consider supporting integration of such structured approaches as part of early palliative care strategies for advanced diseases, thereby improving both quality and equity of care.

Our results showed that although patients with advanced oral cavity cancer in the SOP group received more palliative care consultations, only hospital admissions in the 3 months before death were significantly lower compared with the non-SOP group. However, most other measures of medical resource utilization and invasive treatments remained largely similar between the 2 groups at both 1 month and 3 months before death. This reflects the high complexity of care required for these patients, as they frequently need interventions such as airway management, nutritional support, and pain control, which often necessitate hospitalization.^12,14^ In addition, tertiary cancer centers are often perceived as places for curative treatment rather than comfort-focused care, leading to continued aggressive interventions even when palliative care is involved.^14^ Once intensive treatments begin, patients and families may struggle to shift toward comfort care, particularly if they are unaware of how each decision further commits them to ongoing medical interventions.^7^ Moreover, social and psychological challenges, including poor support systems and high-risk behaviors, can complicate decision-making and care coordination, contributing to sustained hospital-based care.^14,24^ In this context, emerging approaches such as remote monitoring and hospital-based home care may offer additional support by improving symptom control and assisting families, while potentially reducing hospital admissions near the end of life. Future refinement of the SOP model should incorporate these care strategies as part of patients’ treatment options.

Despite increased palliative care consultations in the SOP group, both SOP and non-SOP patients had high rates of DNR documentation. However, even with documented end-of-life preferences, many patients in both groups still underwent invasive procedures.^9^ This highlights the gap between documented preferences and actual care received, suggesting that systemic factors, including hospital culture and entrenched medical mindsets, may continue to drive aggressive interventions.^14^ Similar findings have been reported in studies of the SOP model in pancreatic cancer, where SDM improved advance care planning but did not consistently reduce hospital admissions or the use of intensive interventions.^18^ In our previous work, factors such as relational autonomy, emotional support, and health literacy were identified as influencing whether patients received goal-concordant care at the end of life.^19^ Enhancing the SOP model by incorporating a deeper respect for relational autonomy, strengthening emotional support mechanisms, and improving patient and caregiver health literacy may reduce the likelihood of unwanted invasive procedures in patients with advanced oral cavity cancer. Future studies on the SOP model should incorporate SDM questionnaires to evaluate patients’ perceived involvement in decision-making. This would assess whether the SOP model aligns with the core goals of SDM and may provide insights into addressing the observed gap between high rates of DNR documentation and continued use of invasive treatments.

### Limitations

This study has several limitations. First, a large proportion of participants was still undergoing treatment and was not included in the final analysis, indicating the need for longer follow-up. Although even optimal treatment cannot prevent recurrence in many patients with stage III to IV squamous cell carcinoma of the head and neck, assessing long-term outcomes may require more than 3 years. However, given that the median overall survival for patients with recurrent or metastatic disease is less than 1 year, our 3-year follow-up period remains meaningful. Second, as a single-center study, the findings may not be generalizable to other regions, especially given potential cultural differences in end-of-life decision-making. Expanding the study to include multiple advanced oral cancer treatment centers and international collaborations adopting the SOP model would enhance generalizability. Third, our study did not include measures of quality of life. Incorporating validated instruments such as the EORTC PAL-15 in future research would be valuable to assess whether the SOP model contributes to improvements in patient-reported outcomes as well as care utilization. Fourth, a prospective study, ideally a randomized clinical trial, would provide stronger evidence than this historical cohort comparison design.

## Conclusions

In this cohort study of patients with advanced oral cavity cancer, implementation of the SOP model was associated with greater utilization of multidisciplinary palliative care services. However, despite greater involvement of palliative care services, patients continued to utilize substantial medical resources near the end of life, reflecting ongoing challenges in transitioning from aggressive treatment approaches. These findings underscore the need for improved integration between otolaryngologists and palliative care specialists during the end-of-life phase, as well as clearer communication regarding prognosis and care goals. Strengthening SDM may facilitate greater alignment between treatment plans and patient preferences, ultimately enhancing both quality of life and the quality of end-of-life care. The adoption of the SOP model offers a more proactive and structured framework to support timely and appropriate palliative care delivery for this patient population.

## Notes

Supplement. Data Sharing Statement

## References

[1] Bray F, Laversanne M, Sung H, . Global cancer statistics 2022: GLOBOCAN estimates of incidence and mortality worldwide for 36 cancers in 185 countries. CA Cancer J Clin. 2024;74(3):229-263. doi:10.3322/caac.21834 38572751

[2] Gormley M, Creaney G, Schache A, Ingarfield K, Conway DI. Reviewing the epidemiology of head and neck cancer: definitions, trends and risk factors. Br Dent J. 2022;233(9):780-786. doi:10.1038/s41415-022-5166-x 36369568 PMC9652141

[3] Louie KS, Mehanna H, Sasieni P. Trends in head and neck cancers in England from 1995 to 2011 and projections up to 2025. Oral Oncol. 2015;51(4):341-348. doi:10.1016/j.oraloncology.2015.01.002 25619734

[4] Schenker Y, Arnold RM, Bauman JE, Heron DE, Johnson JT. An enhanced role for palliative care in the multidisciplinary approach to high-risk head and neck cancer. Cancer. 2016;122(3):340-343. doi:10.1002/cncr.29754 26505177

[5] Ullgren H, Kirkpatrick L, Kilpeläinen S, Sharp L. Working in silos? head & neck cancer patients during and after treatment with or without early palliative care referral. Eur J Oncol Nurs. 2017;26:56-62. doi:10.1016/j.ejon.2016.12.003 28069153

[6] Nugent SM, Morasco BJ, Handley R, . Risk of suicidal self-directed violence among US veteran survivors of head and neck cancer. JAMA Otolaryngol Head Neck Surg. 2021;147(11):981-989. doi:10.1001/jamaoto.2021.2625 34617963 PMC8498929

[7] Roscoe LA, Tullis JA, Reich RR, McCaffrey JC. Beyond good intentions and patient perceptions: competing definitions of effective communication in head and neck cancer care at the end of life. Health Commun. 2013;28(2):183-192. doi:10.1080/10410236.2012.666957 22574841

[8] Ferrell BR, Temel JS, Temin S, . Integration of palliative care into standard oncology care: American Society of Clinical Oncology clinical practice guideline update. J Clin Oncol. 2017;35(1):96-112. doi:10.1200/JCO.2016.70.1474 28034065

[9] Huang HL, Tsai JS, Yao CA, Cheng SY, Hu WY, Chiu TY. Shared decision making with oncologists and palliative care specialists effectively increases the documentation of the preferences for do not resuscitate and artificial nutrition and hydration in patients with advanced cancer: a model testing study. BMC Palliat Care. 2020;19(1):17. doi:10.1186/s12904-020-0521-7 32019540 PMC7001377

[10] van den Besselaar BN, Sewnaik A, Hoesseini A, Dorr MC, Baatenburg de Jong RJ, Offerman MPJ. Causes and ways of death in patients with head and neck cancer. JAMA Otolaryngol Head Neck Surg. 2024;150(4):303-310. doi:10.1001/jamaoto.2023.4694 38358760 PMC10870226

[11] van den Besselaar BN, van Hof KS, Sewnaik A, Baatenburg de Jong RJ, Offerman MPJ. Electronic health in the palliative care pathway for patients with head and neck cancer. JAMA Otolaryngol Head Neck Surg. 2025;151(1):19-27. doi:10.1001/jamaoto.2024.3691 39541100 PMC11565372

[12] Mayland CR, Ho QM, Doughty HC, . The palliative care needs and experiences of people with advanced head and neck cancer: a scoping review. Palliat Med. 2021;35(1):27-44. doi:10.1177/0269216320963892 33084497 PMC7797618

[13] Ledeboer QC, Van der Velden LA, De Boer MF, Feenstra L, Pruyn JF. Palliative care for head and neck cancer patients in general practice. Acta Otolaryngol. 2006;126(9):975-980. doi:10.1080/00016480600606764 16864497

[14] Ledeboer QC, Offerman MP, van der Velden LA, de Boer MF, Pruyn JF. Experience of palliative care for patients with head and neck cancer through the eyes of next of kin. Head Neck. 2008;30(4):479-484. doi:10.1002/hed.20733 18023032

[15] Elwyn G, Frosch D, Thomson R, . Shared decision making: a model for clinical practice. J Gen Intern Med. 2012;27(10):1361-1367. doi:10.1007/s11606-012-2077-6 22618581 PMC3445676

[16] Wu YR, Chou TJ, Wang YJ, . Smartphone-enabled, telehealth-based family conferences in palliative care during the COVID-19 pandemic: pilot observational study. JMIR Mhealth Uhealth. 2020;8(10):e22069. doi:10.2196/22069 33021483 PMC7595749

[17] Hanson LC, Zimmerman S, Song MK, . Effect of the goals of care intervention for advanced dementia: a randomized clinical trial. JAMA Intern Med. 2017;177(1):24-31. doi:10.1001/jamainternmed.2016.7031 27893884 PMC5234328

[18] Tseng YL, Lin YC, Hsu WJ, . Shared decision making with oncologists and palliative care specialists (SOP) model help advanced pancreatic cancer patients reaching goal concordant care: a prospective cohort study. Cancer Med. 2023;12(19):20119-20128. doi:10.1002/cam4.6590 37740620 PMC10587919

[19] Lee SC, Shih CY, Chen ST, . Factors contributing to non-concordance between end-of-life care and advance care planning. J Pain Symptom Manage. 2024;67(6):544-553. doi:10.1016/j.jpainsymman.2024.03.004 38479538

[20] World Medical Association. World Medical Association Declaration of Helsinki: ethical principles for medical research involving human subjects. JAMA. 2013;310(20):2191-2194. doi:10.1001/jama.2013.281053 24141714

[21] Elwyn G, Durand MA, Song J, . A three-talk model for shared decision making: multistage consultation process. BMJ. 2017;359:j4891. doi:10.1136/bmj.j4891 29109079 PMC5683042

[22] Joseph-Williams N, Newcombe R, Politi M, . Toward minimum standards for certifying patient decision aids: a modified Delphi consensus process. Med Decis Making. 2014;34(6):699-710. doi:10.1177/0272989X13501721 23963501

[23] Lewis KB, Wood B, Sepucha KR, Thomson RG, Stacey D. Quality of reporting of patient decision aids in recent randomized controlled trials: a descriptive synthesis and comparative analysis. Patient Educ Couns. 2017;100(7):1387-1393. doi:10.1016/j.pec.2017.02.021 28256281

[24] Offerman MP, Pruyn JF, de Boer MF, . Experience of palliative care for patients with head and neck cancer through the eyes of next of kin: impact of an expert center. Head Neck. 2014;36(10):1459-1466. doi:10.1002/hed.23489 23996902

