# Supplementary material for: Shared Decision-Making With Otolaryngologists and Palliative Care Specialists in Oral Cavity Cancer
Source: JAMA Netw Open. 2025 Dec 11;8(12):e2548557. doi: 10.1001/jamanetworkopen.2025.48557 (PMC12699358; doi:10.1001/jamanetworkopen.2025.48557)
Supplement: Supplement. — Data Sharing Statement [file jamanetwopen-e2548557-s001.pdf]

Data Sharing Statement

Huang. Shared Decision-Making With Otolaryngologists and Palliative Care Specialists in Oral Cavity Cancer. *JAMA Netw Open*. Published December 11, 2025.
doi:10.1001/jamanetworkopen.2025.48557

Data
Data available: Yes
Data types: Deidentified participant data
How to access data: tennishuang@gmail.com
When available: With publication

Supporting Documents
Document types: None

Additional Information
Who can access the data: researchers whose proposed use of the data has been approved
Types of analyses: for research
Mechanisms of data availability: with investigator support
